# The inflammation-depression link: How social networks buffer or exacerbate risk

**DOI:** 10.1016/j.bbih.2025.101052

**Published:** 2025-07-04

**Authors:** Nur Hani Zainal

**Affiliations:** National University of Singapore, Department of Psychology, Kent Ridge Campus, Singapore

**Keywords:** Depression, Inflammation, Longitudinal, Moderator, Social support

## Abstract

**Aims:**

Major depressive disorder (MDD) is a prevalent mental disorder, and low social support and high strain could impact its long-term symptom severity. Increased inflammation, marked by C-reactive protein (CRP) and fibrinogen, has also been correlated with more MDD symptoms. However, the inflammation-MDD symptom association might vary by social support dimensions. The current study thus examined how social support dimensions moderated the inflammation-MDD severity correlation.

**Methods:**

Community adults (*N* = 1,054) with and without MDD provided plasma samples to measure CRP and fibrinogen levels and completed self-reports of perceived support and strain from family, friends, and partners at Wave 1 (W1). MDD severity was assessed at W1 and Wave 2 (W2, nine-year follow-up). Multiple linear regressions and generalized additive modeling (GAM) assessed how W1 social support dimensions and inflammation levels interacted to predict W2 MDD severity, controlling for clinical and sociodemographic covariates.

**Results:**

Increased W1 fibrinogen predicted higher W2 MDD severity in participants with lower (vs. higher) W1 social support and higher (vs. lower) social strain (|standardized β| = 0.18–2.31 vs. 0.01–0.03). Further, increased CRP predicted more MDD symptoms in participants with higher (vs. lower) social strain (|β| = 0.24–0.26 vs. 0.15–0.16). These significant interaction findings were identical in linear and GAM models that accommodate non-linear associations. **Conclusions**: Results suggested that increased proinflammatory activity indexed by CRP and fibrinogen levels could predict nine-year MDD severity under social strains, consistent with the social signal transduction theory. Improving social support and decreasing social strain might buffer inflammation-related depression.

## Highlights

• Social strain was linked to greater nine-year inflammation-MDD severity connection.
• Fibrinogen interacted with social support and strain in predicting nine-year MDD.
• CRP levels interacted with social strain in predicting long-term MDD severity.
• GAM approach replicated similar linear and non-linear variable associations.
• The social signal transduction theory might inform MDD prevention approaches.

Major depressive disorder (MDD) is a common mental disorder with global 12-month prevalence estimates ranging from 11 to 28 % in community samples (Chen et al., 2012; Vandeleur et al., 2017) and 12 to 36 % in clinical populations (Hunt et al., 2020b; Kuhlmann et al., 2019). Heightened MDD symptoms, such as anhedonia, depressed mood, and related psychiatric comorbidities, have been reliably linked to functional impairments in epidemiological surveys across diverse countries (Roest et al., 2021). These functional impairments could encompass issues related to career development (Oenning et al., 2018), physical health (Berk et al., 2023), and social relationships (Bird et al., 2018). Therefore, identifying the distal risk factors, defined as central preconditions that raise disease risk over time, of MDD is crucial.

Proinflammatory processes have been considered critical to the etiology of increased MDD symptom severity across long durations. Although pro-inflammatory processes encapsulate a wide array of inflammation markers, two salient ones relevant to this study are C-reactive protein (CRP) and fibrinogen. CRP represents an acute-phase protein that the liver produces in response to inflammation (Felger et al., 2020). Fibrinogen is a coagulant (i.e., blood clotting agent) that similarly initiates pro-inflammatory processes (Pieters and Wolberg, 2019). The cytokine model of depression (Koo et al., 2017) posits that increased fibrinogen and CRP would predict more MDD symptoms over lengthy periods via several plausible pathways. Putative pathways or mechanisms include deviations and dysfunctions in the brain's endocrine, metabolism, and neuroplastic systems (Horowitz and Zunszain, 2015). For example, chronic increased peripheral inflammation could dysregulate or suppress the hypothalamic-pituitary-adrenal (HPA) axis pathways. This process may result in suboptimal cortisol secretion, signaling, and glucocorticoid resistance, which can exacerbate MDD severity, including attentional and motivational issues over prolonged periods (Hassamal, 2023). Serotonin levels may also be depleted through excessive proinflammatory processes in the kynurenine pathway, which involves altering the indoleamine-2,3-dioxygenase (IDO) enzyme and breaking down tryptophan over extended durations (Hunt et al., 2020a). Proinflammatory markers (e.g., fibrinogen) may also compromise the structural integrity of the blood-brain barrier, thereby increasing its permeability (Futtrup et al., 2020). Increased blood-brain barrier permeability may trigger the entry of these proinflammatory markers into the central nervous system (CNS), thereby magnifying stress reactivity and MDD severity over time (Medina-Rodriguez and Beurel, 2022).

Consistent with these theories, a recent prospective study (Zainal and Newman, 2021) and meta-analysis (Mac Giollabhui et al., 2021) of longitudinal studies on the bidirectional connections between inflammation and depression revealed consistent evidence that increased CRP and fibrinogen predict future MDD symptoms. However, these studies also suggested that the pathway from proinflammatory processes to future increased MDD severity is highly complex and variable. The high variability in the inflammation-MDD pathway raises a question about potential moderators. Which person with higher inflammation levels is most likely susceptible to increased MDD many years later?

Social support and strain might be essential candidate moderators in linking proinflammatory activity to higher future MDD severity. Social support is defined as the subjective accessibility to affection, care, and understanding from individuals’ social networks, encapsulating emotional and tangible help from family, friends, or partners (Taylor, 2011). Social strain is referred to as the adverse aspects of interpersonal exchanges, such as conflict, disparagements, or excessive requests (Rook, 1984). Lower support and higher strain might worsen the effects of proinflammatory activity on future MDD symptoms, as the buildup of stressful interpersonal experiences might chronically reinforce suboptimal coping responses and stress reactivity (Slavich and Irwin, 2014). These joint interactive pathways would likely unfold, given how strong social networks, positive relations with others, and fewer social strains (e.g., interpersonal conflicts) generate protective effects in modulating inflammatory processes (cf. social signal transduction theory of depression; Quinn et al., 2020). Overall, participants facing increased social strain – characterized by overly critical, frustrating, and inconsistent interactions in their social circles – may be at risk of the negative impacts of elevated CRP and fibrinogen levels on future MDD severity. Moreover, individuals with higher emotional support, indicating greater accessibility to empathy and help from essential relationships, could display more resilience against the adverse effects of proinflammatory activity. Results highlight the importance of both positive (social support) and negative (social strain) dimensions of social relationships interacting with inflammation levels to predict long-term MDD severity.

Indirect support has been observed for these propositions on the moderation effect of social support on inflammation-depression pathways. First, increased proinflammatory activity mediated the pathway from lower social support to higher depressive symptoms in breast cancer survivors (Hughes et al., 2014). Second, better social support indicators conferred protective effects against future increases in MDD symptoms among community adults, even after adjusting for demographic and lifestyle confounds (Yang et al., 2014). Relatedly, a quantitative synthesis of 41 cross-sectional and longitudinal reports showed robust negative associations between social support factors and proinflammatory marker levels (Uchino et al., 2018). Finally, people who experienced early-life stressors self-reported higher depression as adults, but only if they jointly encountered fewer social exchanges and perceived lower social support (Nakamura et al., 2022). Together, these outcomes suggest that the frequency of social exchanges, perceived social support, and strain may buffer or mitigate the long-term adverse impacts of increased inflammation on future MDD severity.

The present study thus determined the moderating role of social support dimensions—support and strain—on inflammation-MDD pathways and built on prior studies in several ways. First, previous studies mostly assumed linear relations among inflammation, social support, and MDD. However, prior research suggested that the associations among these variables might be non-linear (Eisman et al., 2015; Zhai et al., 2022). Such non-linearities emphasize the importance of using advanced modeling techniques, such as generalized additive modeling (GAM; Wood et al., 2015), to capture complex non-linear patterns. GAM refers to statistical approaches that expand on generalized linear modeling techniques by permitting non-linear associations between predictor or moderator variables and the outcome variable via smooth function applications (Wieling, 2018). GAMs are appropriate for analyzing non-linear relations among key variables of interest in the present study (refer to Chen et al., 2016; Henneghan et al., 2021; Lee et al., 2023, for relevant examples). Second, most studies examining the associations among inflammation, social support, and MDD severity have been cross-sectional (Colasanto et al., 2020; Rueger et al., 2016), limiting the ability to draw weak causal inferences (Pearl, 2014). Third, prior studies on this topic have focused on one aspect of social support, although previous research has highlighted that social strain may be a more potent predictor of inflammation than social support (e.g., Yang et al., 2014).

Thus, the current study investigated how social support and social strain dimensions moderated the associations of increased CRP and fibrinogen levels with future MDD severity. Given theory and research, we hypothesized that higher fibrinogen levels would predict more future MDD symptoms, especially in individuals with lower social support and higher social strain (Hypothesis 1). The same moderation pattern was also anticipated but with CRP levels in the multivariate model (Hypothesis 2). Moreover, these patterns were expected to hold across both hypotheses in the context of linear and non-linear relations. This expectation remained after adjusting for various potential demographic and clinical confounds.

## 1 Method

### 1.1 Participants

Community adults (*N* = 1,054) from the general population participated in the Midlife Development in the United States (MIDUS) study in two waves (Wave 1; W1; Wave 2; W2; Ryff et al., 2019a; Ryff et al., 2017; Ryff et al., 2019b). At W1, they had an average age of 55.19 years (*SD* = 11.81, range = 25–74). Regarding self-reported gender, 45.3 % (477/1054) identified as men, and 54.7 % (577/1054) identified as women. Education levels were diverse, with 44.1 % (465/1054) holding a college or university degree and above, 22.6 % (238/1054) having a high school education, and the remaining 33.3 % (351/1054) having no high school education or declining to disclose. Regarding diagnostic status, 11.70 % (123/1054) had MDD at W1, and 9.11 % (96/1054) at W2. Table S1 in the online supplemental materials (OSM) offers more descriptive statistics, including relevant percentages and subgroup breakdowns.

### 1.2 Procedures

The present study included participants who underwent the procedures of the MIDUS W1 Biomarker study (Weinstein et al., 2019), as this approach directly addressed the research aims. Participants completed clinical interviews assessing the diagnosis and severity of MDD symptoms at W1 and W2, and self-reports assessing perceived social support at W1. Further, they provided biomarker data at W1, which included proinflammatory markers.

### 1.3 Measures

**W1 and W2 MDD symptoms.** MDD symptoms were assessed using the World Health Organization's Composite International Diagnostic Interview-Short Form (CIDI-SF; Kessler et al., 1998), which was based on the Diagnostic and Statistical Manual of Mental Disorders, Revised Third Edition (DSM-III-R; American Psychiatric Association, 1987). The presence of these seven MDD symptoms was recorded during the clinical interview: anhedonia (loss of interest in pleasurable activities), appetite disturbances, concentration issues, fatigue, low self-worth, suicidal ideation, and sleep difficulties. Internal consistency values (Cronbach's α = .927 and .930 at W1 and W2, respectively) were good in the present study. MDD symptom scores could range from 0 (*lowest severity*) to 7 (*highest severity*).

**W1 Perceived social** support**.** A 12-item MIDUS-specific measure of social support was administered to assess perceived social support (Guevara and Murdock, 2019). Participants self-reported the degree to which their family members, friends, and partner or spouse offered affection and care toward them. Examples of items included assessing the extent to which these significant others cared for and understood the participants, as well as how the participants could rely on and confide in them. Item scores ranged from 1 (*not at all*) to 4 (*a lot*). The same four items were repeated across the three unique social support figures. Total scores theoretically ranged from 14 to 56. This scale has been shown to have good internal consistency (Cronbach's α = .787 herein) and construct validity (Creaven et al., 2020; Elliot et al., 2018).

**W1 Perceived social strain.** Another 12-item self-report developed by the MIDUS researchers was administered to measure perceived social strain (Kane and Krizan, 2021). Participants also self-reported the extent to which their family members, friends, and partner or spouse were excessively critical, demanding, and disappointing, and behaved in ways that angered them. The same four items were again repeated across the three distinct social strain figures. Similar to the social support dimension, item scores varied from 1 (*not at all*) to 4 (*a lot*), and total scores could range from 14 to 56. This scale had good internal consistency (α = .776), retest reliability, and construct validity (Fitzgerald and Morgan, 2022; Teo et al., 2013).

**W1 and W2 Proinflammatory markers.** Participants had blood samples drawn after fasting overnight and before breakfast on the second day of the biomarker protocol, allowing for the collection of plasma assays to evaluate proinflammatory markers (Dienberg Love et al., 2010). Whole blood samples were stored in a -65 °C freezer until these proinflammatory markers were assessed at another laboratory using standardized protocols. These blood plasma samples were drained into tubes containing anticoagulants to prevent blood clotting before undergoing centrifugation procedures that separated the blood plasma. The MIDUS researchers used a BNII nephelometer with the blood plasma samples to measure levels of CRP (cf. particle-improved immunonephelometric assay) and fibrinogen (N Antiserum to Human Fibrinogen developed by Dade Behring Inc. in Deerfield, IL; Ospina et al., 2022). Regarding analytic methods used, the BNII nephelometer quantifies CRP and fibrinogen levels by forming immune complexes between the target analyte and unique antibodies (Witzel et al., 2025). Immune complexes are antigen-antibody groups that trigger immune responses, but unique antibodies are B-cell-generated proteins that choose distinct antigens (foreign biomarkers in the immune system; Mahendra et al., 2022; Marshall et al., 2018). This process triggers alterations in light scattering, which are captured to assess the analyte's concentration levels. These biomarker assay procedures were done at the University of Vermont (Burlington, VT) to maximize measurement consistency and precision.

### 1.4 Data analysis

All analyses were done using *RStudio* (R Core Team, 2024). Random forest imputation was used to handle missing data with the *missRanger* package, which was observed in 7.4% of the entire data set (Mayer, 2024). Random forest imputation was chosen over parametric methods, such as multiple imputation, due to its ability to accommodate potential complex interactions and nonlinear relations among variables (Stekhoven and Buhlmann, 2012) and to stay robust when the data was missing not at random (MNAR; Tang and Ishwaran, 2017). As shown in Table S2 in the OSM, dropouts (*n* = 178) were significantly older, more likely to identify as White, had higher baseline social strain, and exhibited higher fibrinogen levels compared to completers (*n* = 876). These between-group differences rendered the dataset's missingness pattern MNAR, making random forest imputation an appropriate method for handling missing data in this context. Other model assumptions of multiple linear regression were examined and aligned with expectations based on standard diagnostic tests of homoscedasticity, independent residual variances, linearity, multicollinearity, and multivariate normality (Hainmueller et al., 2018; Karazsia et al., 2014). Following random forest imputation and these preprocessing checks, the analytic sample size was consistently 1,054 for all linear and GAM models described below, with 123 participants having MDD symptoms at W2.

Multiple linear regression was employed to test the impacts of one predictor (W1 CRP or fibrinogen levels), two moderators (W1 social support and strain), and two two-way interactions (inflammation × social support and inflammation × social strain) on W2 MDD severity. W1 MDD severity was added as a covariate in all analyses. This method enabled the assessment of both main effects and plausible interactions, aligning with the research aims (Irwin and McClelland, 2001; Shieh, 2010). By including possible moderator effects in the multivariate equation, the analysis could identify how the impact of a predictor may vary based on the level of a moderator, thereby enriching the understanding of prospective variable associations (Aguinis and Gottfredson, 2010; Yuan et al., 2014).

Sensitivity analyses were conducted with GAM using the *mgcv* (Wood, 2017) package. GAMs were employed to investigate potential non-linear relationships between proinflammatory markers (CRP and fibrinogen) and social support dimensions (support and strain) in predicting nine-year MDD severity. GAMs are more flexible than standard linear regression models, as they capture potentially complex, data-driven, non-linear relationships between predictor and outcome variables. Specifically, the smooth function *s*() was applied to accommodate possible non-linearities in both main effects and two-way interactions of the proinflammatory marker and social support or strain variables (Imai et al., 2010; VanderWeele and Tchetgen Tchetgen, 2017). The Restricted Maximum Likelihood estimator was used, and model diagnostics were generated (Wood, 2011). To this end, this GAM sensitivity analysis enhances rigor by testing potential non-linearities and facilitating replication attempts (Loh et al., 2022; Wood, 2004). If both GAMs and linear regression models generated identical patterns, the robustness of the results was reinforced by showing that variable associations align across distinct modeling methods.

To ease interpretations, Cohen's *d* effect sizes were computed with the *d* = 2*t*/√(*df*) equation (Rosenthal, 1994), where *t* is the *t*-statistic of the parameter estimate, and *df* is the model's degrees of freedom. Cohen's *d* differs from standardized regression estimates (β; Cohen et al., 2003), which were computed and reported throughout, as well as the unstandardized regression beta weights (*b*). Although standard guidelines set Cohen's *d* values of 0.2, 0.5, and 0.8 as small, moderate, and large, respectively, even small *d* values of 0.1 could have important public health implications, especially with large datasets spanning over long intervals, such as nine years (Funder and Ozer, 2019).

The present analyses also tested whether the observed effects remained statistically significant after including various covariates in a series of sensitivity analyses. Regarding sociodemographic variables, we tested age (Straka et al., 2020), gender (Niles et al., 2018), education level (Krogh et al., 2014), and race (Toussaint et al., 2022) as covariates. With respect to psychopathology variables, we assessed whether similar outcomes emerged when including childhood trauma (Kleih et al., 2022), generalized anxiety disorder (GAD; Zainal and Newman, 2022b), and panic disorder symptoms (Choi et al., 2021) as covariates. We also tested if proinflammatory markers moderated the pathway from social support to future MDD symptoms. Parameter estimates with *p*-values of < 0.05 were considered statistically significant.

## 2 Results

### 2.1 Interaction between W1 fibrinogen and social support dimensions predicting W2 MDD severity (Hypothesis 1)

To test Hypothesis 1, we specified W1 fibrinogen levels as the predictor and W2 MDD severity as the outcome, adjusting for W1 MDD severity. W1 social support and strain dimensions were simultaneously tested as moderators. Thus, the model contained four main effects and two two-way interactions between social support and fibrinogen as well as between social strain and fibrinogen levels.

**Fig. 1:**
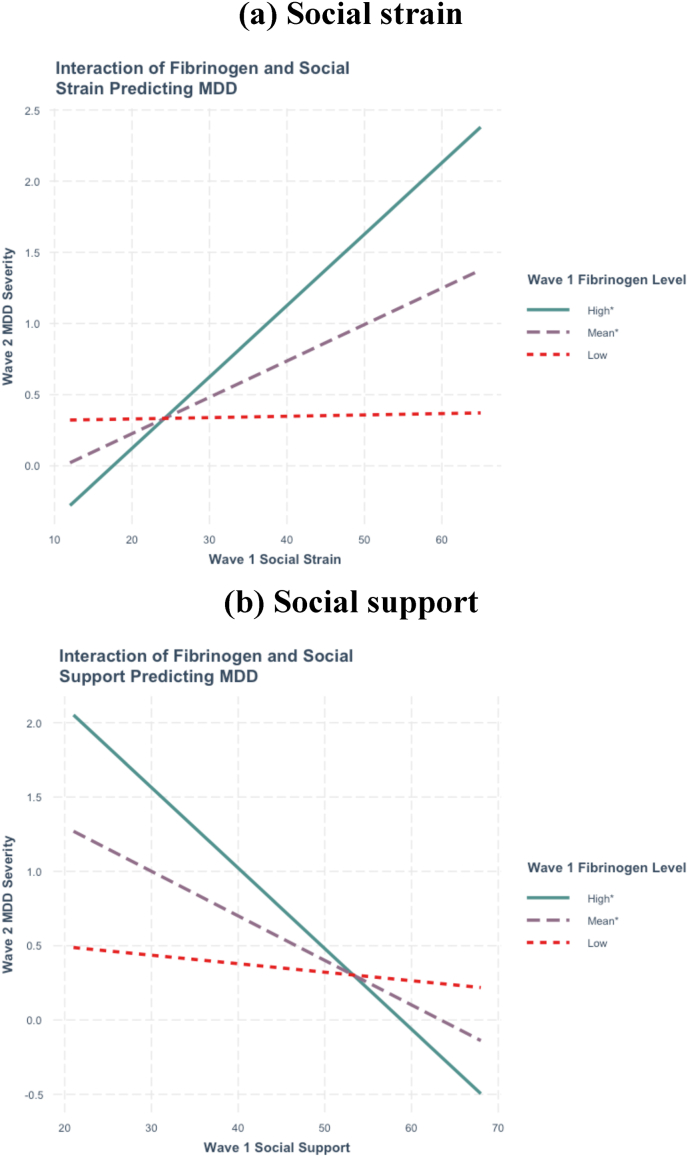
W1 Social support dimensions predicting W2 MDD severity by W1 fibrinogen levels (Linear models) (N = 1,054) *Note.* W1, wave 1; W2, wave 2; MDD, major depressive disorder. An asterisk in the legend means that the simple slope of the association between W1 social support or strain and W2 MDD severity was statistically significant at that level.

Fibrinogen levels significantly interacted with social support (β = −1.90, *d* = −0.20, *p* = .001) and social strain (β = 2.31, *d* = 0.26, *p* < .001) in predicting future MDD severity (Table 1(a)). Mean fibrinogen levels were defined as log-transformed values between 4.78 and 5.64, with high levels as ≥ 5.64 and low levels as ≤ 4.78. Simple slope analysis showed that the association between higher social strain predicting greater future MDD severity was significant at the *mean* (β = 0.19, *d* = 0.46, *p* < .001) and *high* (β = 0.37, *d* = 0.65, *p* < .001) fibrinogen levels but not at *low* (β = 0.01, *d* = 0.01, *p* = .910) fibrinogen levels (Fig. 1(a)). In addition, the relationship between lower social support predicting higher future MDD severity was significant at the *mean* (β = −0.18, *d* = −0.44, *p* < .001) and *high* (β = −0.32, *d* = −0.55, *p* < .001) fibrinogen levels instead of at *low* (β = −0.03, *d* = −0.06, *p* = .590) fibrinogen levels (Fig. 1(b)). Sensitivity analyses revealed that these findings remained similar when examining non-linear associations (Table 1(b)).

**Table 1:** Multiple regression analysis of W1 fibrinogen levels interacting with W1 social support dimensions to predict W2 MDD symptom severity (N = 1054).

Parameter estimate	*b*	*(SE)*	*t*	*p*	*d*
**(a) Linear model estimates**					
Intercept	−5.602	(3.084)	−1.816	.070	−0.112
W1 MDD severity	0.275	(0.027)	10.207	.000	0.631
W1 Social support	0.264	(0.092)	2.880	.004	0.178
W1 Fibrinogen	1.258	(0.589)	2.135	.033	0.132
W1 Social strain	−0.273	(0.070)	−3.867	.000	−0.239
W1 Social support x W1 Fibrinogen	−0.056	(0.018)	−3.217	.001	−0.199
W1 Social strain x W1 Fibrinogen	0.057	(0.013)	4.246	.000	0.262
*R*^*2*^	15.2 %				
Adjusted *R*^*2*^	14.7 %				
*F*-statistic	31.336				
*p*	0.000				
**(b) GAM non-linear estimates**					
Intercept	0	(0.000)	–	–	–
W1 MDD severity	0.272	(0.027)	10.168	<.001	0.628
W1 Social support	−0.314	(0.291)	−1.077	.282	−0.067
W1 Fibrinogen	−0.018	(0.028)	−0.653	.514	−0.040
W1 Social strain	0.093	(0.028)	3.322	.001	0.205
W1 Social support x W1 Fibrinogen	8.416	(11.277)	2.079	.021	0.128
W1 Social strain x W1 Fibrinogen	6.914	(9.683)	2.748	.002	0.170
Rank	59/62				
Adjusted *R*^*2*^	0.178				
Deviance Explained	19.20 %				

*Note.* W1, wave 1 (2004–2006); W2, wave 2 (2013–2014); MDD, major depressive disorder; GAM, generalized additive models; REML, restricted maximum likelihood. At W2, 123 participants exhibited at least one symptom of MDD.

### 2.2 Interaction between W1 CRP and social support dimensions predicting W2 MDD severity (Hypothesis 2)

To test Hypothesis 2, we specified W1 CRP levels as the predictor and W2 MDD severity as the outcome, controlling for W1 MDD severity. W1 social support and strain dimensions were concurrently examined as moderators. Therefore, this model comprised four main effects and two two-way interactions between social support and CRP levels as well as between social strain and CRP levels.

**Fig. 2:**
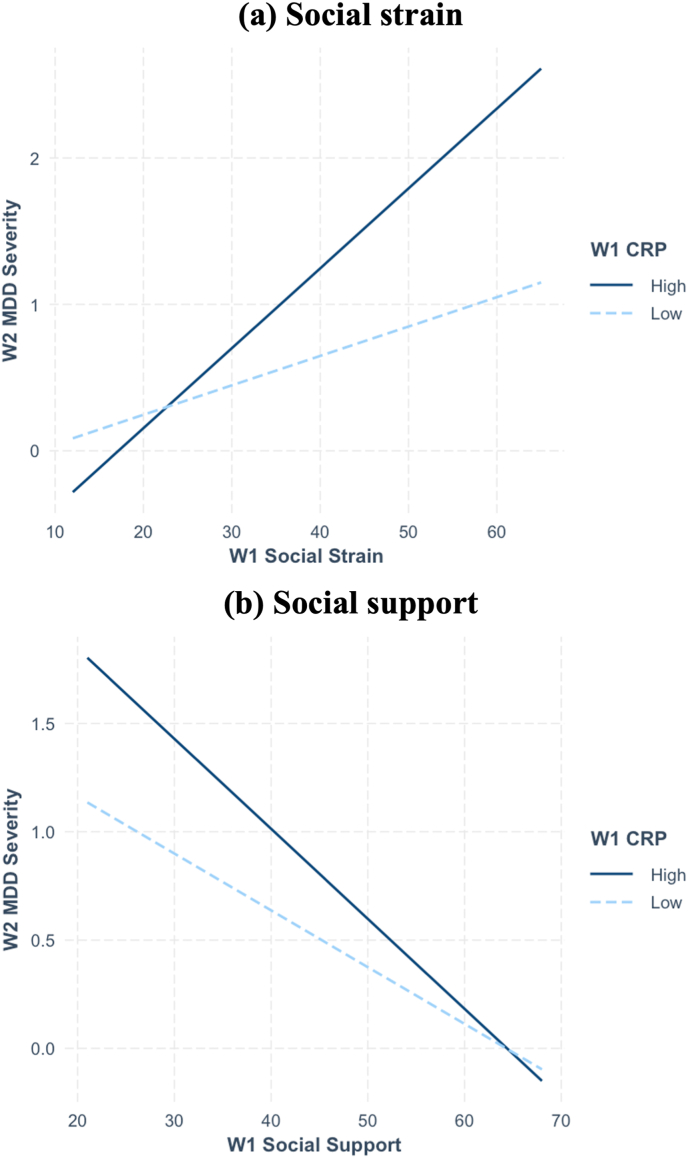
W1 Social support dimensions predicting W2 MDD severity by W1 CRP levels (Linear models) (N = 1,054) *Note.* W1, wave 1; W2, wave 2; MDD, major depressive disorder; CRP, C-reactive protein. An asterisk in the legend means that the simple slope of the association between W1 social support or strain and W2 MDD severity was statistically significant at that level.

CRP levels significantly interacted with social strain (β = 0.26, *d* = 0.15, *p* = .019), but not social support (β = −0.16, *d* = −0.05, *p* = .415) in predicting future MDD severity (Table 2(a)). High CRP levels were defined as log-transformed values ≥ 0.55, and low CRP levels as 0. Simple slope analysis demonstrated that the relationship between greater social strain predicting higher future MDD severity was significantly more potent at *high* CRP levels (β = −0.24, *d* = 0.36, *p* < .001) than at *low* CRP levels (β = −0.15, *d* = 0.27, *p* < .001; Fig. 2). The pattern of findings remained similar when accounting for non-linear associations (Table 2(b)).

**Table 2:** Multiple regression analysis of W1 CRP levels interacting with W1 social support dimensions to predict W2 MDD symptom severity (N = 1054).

Parameter estimate	*b*	*(SE)*	*t*	*p*	*d*
**(a) Linear model estimates**					
Intercept	0.904	(0.273)	3.317	.001	0.205
W1 MDD severity	0.273	(0.027)	10.031	.000	0.620
W1 Social support	−0.026	(0.008)	−3.228	.001	−0.200
W1 CRP	−0.062	(0.653)	−0.095	.924	−0.006
W1 Social strain	0.020	(0.007)	3.075	.002	0.190
W1 Social support x W1 CRP	−0.015	(0.019)	−0.816	.415	−0.050
W1 Social strain x W1 CRP	0.034	(0.015)	2.353	.019	0.145
*R*^*2*^	13.9 %				
Adjusted R^2^	13.4 %				
*F*-statistic	28.207				
*p*	0.000				
**(b) GAM non-linear estimates**					
Intercept	–	–	–	–	–
W1 MDD severity	0.273	(0.027)	10.168	<.001	0.631
W1 Social support	–	–	–	–	–
W1 CRP	−0.052	(0.017)	−3.011	.003	−0.187
W1 Social strain	0.091	(0.027)	3.428	.001	0.213
W1 Social support x W1 CRP	9.308	(12.124)	1.783	.040	0.111
W1 Social strain x W1 CRP	6.251	(8.818)	3.036	.002	0.188
Rank	59/62				
Adjusted *R*^*2*^	0.172				
Deviance Explained	18.60 %				

*Note.* W1, wave 1 (2004–2006); CRP, C-reactive protein; W2, wave 2 (2013–2014); MDD, major depressive disorder; GAM, generalized additive models; REML, restricted maximum likelihood. Any ‘–’ indicates that the specific parameter values could not be estimated. At W2, 123 participants exhibited at least one symptom of MDD.

### 2.3 Sensitivity analyses

To reiterate, we examined if the pattern of statistically significant findings observed in the initial moderation models remained after adjusting for age, gender, race, education, childhood trauma, GAD, and panic disorder symptoms as covariates. When CRP levels were the predictor, the moderation analyses produced similar outcomes regarding effect size magnitude and direction, as well as statistical significance thresholds. The same pattern also occurred when the moderation analyses examined fibrinogen levels as the predictor (Table S3–S16 in the OSM). Collectively, the present findings were fully aligned with the study hypotheses when testing fibrinogen levels as the predictor (Hypothesis 1). However, the results were partially consistent with expectations when examining CRP levels as the predictor (Hypothesis 2).

## 3 Discussion

The current study examined how social support and strain moderated the longitudinal relationships between baseline levels of the proinflammatory markers CRP and fibrinogen and nine-year MDD severity. The findings were partially consistent with the study's hypotheses. Social strain notably interacted with both increased CRP and fibrinogen levels to predict higher nine-year MDD severity. However, lower social support substantially interacted with fibrinogen levels, but not CRP levels. These moderation effects were strongest at moderate-to-high, rather than low, proinflammatory activity levels and remained after controlling for several clinical and demographic covariates. Overall, the results suggested that social support could either buffer or exacerbate the relationship between increased inflammation and future MDD symptom severity; however, this relationship varied depending on the proinflammatory marker and specific social support dimensions.

The notable interaction between social support dimensions and fibrinogen levels as a predictor of MDD severity nine years later highlighted the complex interplay among these risk and protective factors. Whereas increased fibrinogen accentuated the long-term effects of higher social strain and lower social support on future MDD severity, these patterns did not survive (i.e., became statistically insignificant) at low fibrinogen levels. This moderation effect might imply that interpersonal stressors could amplify the impact of increased fibrinogen levels on MDD symptoms over a protracted timescale. Increased fibrinogen levels have been linked to neuroinflammatory pathways, such as activating microglia and compromising neurogenesis and neuroplasticity, which might heighten future MDD severity (Patel et al., 2024; Petersen et al., 2018). Moreover, increased fibrinogen might impact the permeability of the blood-brain barrier, plausibly expanding the entryways of low-grade peripheral chronic proinflammatory activity into the CNS (Golanov et al., 2019; Piers et al., 2018). Together, these plausible pathways and sequelae might render people vulnerable to the adverse impacts of low social support and high social strain, raising the risk of increased nine-year MDD severity.

A similar pattern was observed when CRP levels were included in the equation, but the interaction was substantial with social strain instead of social support. The greater effect of social strain than support might be due to the fact that negative social experiences tend to be recalled more strongly than positive ones, a phenomenon rooted in evolutionary psychology (Yang et al., 2014). Another account might involve the mediating role of perceived stress triggered by conflicts, criticisms, and demands from the social circle of individuals (Acoba, 2024). Increased CRP levels have been related to more depression symptoms, especially among persons facing high social stressors or strains (Orsolini et al., 2022; Yang et al., 2014), implying a potentially synergistic impact between this proinflammatory marker and social strain on future MDD severity. Based on prior research (Iob et al., 2020; Lei et al., 2025), hyperactivation of the HPA axis, which may trigger suboptimal cortisol modulation, could mediate the interaction between social strain and CRP levels in the development of MDD symptoms over time. Further, increased CRP levels have been correlated with unique depression clusters or symptoms, including low energy and psychomotor aberrations (Franklyn et al., 2022; Moriarity et al., 2021). Such patterns suggest that higher CRP levels, coupled with social strain, could be key risk factors for elevated MDD symptoms. These conjectures require further testing in future research employing longitudinal designs.

Moreover, the alignment of findings when testing linear and non-linear models underscores the importance of sensitivity analyses to confirm the robustness of the complex relationships among inflammation, social support, and future MDD severity. Such concordance increases confidence that the results are probably reliable and replicable, pending more similar studies. Their agreement suggests that the links between CRP or fibrinogen levels and future MDD symptoms may vary based on analytical, interpersonal, and measurement factors (Lee and Whooley, 2023; Luan et al., 2020; Zainal and Newman, 2023), although this warrants further exploration.

The limitations of the present study warrant attention. First, we recognized that proinflammatory processes encompass many biomarkers; however, we focused on CRP and fibrinogen due to their importance and similarity. CRP is a well-established low-grade, peripheral, systemic proinflammatory activity marker and has been linked to depression (Fatemian et al., 2024), rendering it a critical biomarker to explore as a distal risk factor of high MDD severity. Fibrinogen, another acute-phase reactive protein crucial in proinflammatory processes, has been reliably associated with depression (Wium-Andersen et al., 2013), highlighting its importance in understanding the interaction between inflammation and social support or strain on future MDD severity. Although other markers were available (e.g., intracellular adhesion molecule [ICAM-1]) in the MIDUS data set, we focused on CRP and fibrinogen due to their similarities as key acute-phase reactive proteins and distinct proinflammatory activity mechanisms (Kushner and Mackiewicz, 2020). Future prospective replication studies should test distinct pro-inflammatory markers, such as interleukin-12 and interleukin-18 (Osimo et al., 2020). Second, replication attempts should utilize measures aligned with the current DSM-5 diagnostic taxonomy (American Psychiatric Association, 2022) as the 18-year MIDUS project employed the prior DSM-III-R version of the CIDI-SF for consistency across time points. Third, associations between inflammation and MDD symptoms are probably reciprocal (Mac Giollabhui et al., 2021). However, our research question focused on studying how social support dimensions might alter the sign and strength of distal risk factors (increased CRP and fibrinogen) that predict MDD. Future research could apply the scar theory (MDD symptoms predicting future pro-inflammatory markers; Zainal and Newman, 2021, 2022a, 2023) to investigate how social support dimensions fit into the equation and provide a more nuanced understanding of this topic. Fourth, future studies should investigate the role of behavioral genetics and related unexplored and unmeasured third variables (Sforzini et al., 2023; Zavos et al., 2022).

Despite these limitations, the current study had some strengths. It employed a longitudinal design spanning a nine-year period, offering insights into how specific proinflammatory activity, social support, and mental health factors interact with one another from middle to older adulthood. Various sensitivity analyses were also conducted to ensure the robustness of observations.

If these patterns were replicated, some clinical implications would merit consideration. As individuals facing high social strain are vulnerable to the adverse effects of increased CRP and fibrinogen levels, improving social support dimensions and reducing proinflammatory activity could prevent future elevated MDD severity. Decreasing low-grade, proinflammatory CRP and fibrinogen activity through enhanced physical activity and the adoption of anti-inflammatory diets (e.g., foods enriched in omega-3 fatty acids) may better regulate neuroinflammatory pathways associated with depression. Simultaneously, boosting social support and reducing social strain might improve mood regulation, thereby decreasing CRP and fibrinogen levels and reducing depressive symptoms. Future public health research should investigate how reducing CRP, fibrinogen, and related proinflammatory activity while improving social support dimensions might be promising pathways to decrease the long-term risk of heightened MDD severity.

Regarding potential future applications, clinicians and policymakers could promote lifestyle improvements for the broader population, such as consuming more Mediterranean-style meals (Altun et al., 2019; Milaneschi et al., 2011) and engaging in regular physical activity in various settings (Frank et al., 2019; Ignacio et al., 2019). Multifaceted programs have been effective in enhancing social support and reducing social strain. These facets may include consciousness-raising efforts, family systems interventions, peer support programs, and phone or web-based interventions for middle-aged to older adults (Dam et al., 2016; Orazani et al., 2023). Based on precision mental health (Lutz et al., 2024), implementation science (Beidas et al., 2025), and the present findings, clinical science might profit from identifying which strategies work best for whom based on CRP and fibrinogen levels as well as social support and strain to possibly enhance preventive care.

## Availability of data and materials

Data from this study can be obtained from an online repository, the Inter-university Consortium for Political and Social Research (ICPSR; W1 clinical interview and self-report dataset: https://tinyurl.com/icpsr4652; W1 biomarker dataset: https://tinyurl.com/icpsr29282; W3 clinical interview and self-report dataset: https://tinyurl.com/icpsr36346). More details on the uniqueness of this study compared to other studies that used the MIDUS dataset can be found in the OSM.

## Funding

The present meta-analysis received funding from the 10.13039/501100001352National University of Singapore (NUS) Presidential Young Professorship (PYP) Start-Up Grant and White Space Fund. All funding agencies are not responsible for the analyses or interpretations presented here.

## Declaration of competing interest

The authors declare that they have no known competing financial interests or personal relationships that could have appeared to influence the work reported in this paper.

## Data Availability

The author does not have permission to share data.

## References

[bib1] Acoba E.F. (2024). Social support and mental health: the mediating role of perceived stress. Front. Psychol..

[bib2] Aguinis H., Gottfredson R.K. (2010). Best‐practice recommendations for estimating interaction effects using moderated multiple regression. J. Organ. Behav..

[bib3] Altun A., Brown H., Szoeke C., Goodwill A.M. (2019). The mediterranean dietary pattern and depression risk: a systematic review. Neurol. Psychiatr. Brain Res..

[bib4] American Psychiatric Association (1987).

[bib5] American Psychiatric Association (2022). https://psychiatryonline.org/doi/book/10.1176/appi.books.9780890425787.

[bib6] Beidas R.S., Boyd M., Casline E., Scott K., Patel-Syed Z., Mills C., Becker S.J. (2025). Harnessing implementation science in clinical psychology: past, present, and future. Annu. Rev. Clin. Psychol..

[bib7] Berk M., Kohler-Forsberg O., Turner M., Penninx B., Wrobel A., Firth J., Marx W. (2023). Comorbidity between major depressive disorder and physical diseases: a comprehensive review of epidemiology, mechanisms and management. World Psychiatry.

[bib8] Bird T., Tarsia M., Schwannauer M. (2018). Interpersonal styles in major and chronic depression: a systematic review and meta-analysis. J. Affect. Disord..

[bib9] Chen H.H., Hwang F.M., Lin L.J., Han K.C., Lin C.L., Chien L.Y. (2016). Depression and social support trajectories during 1 year postpartum among marriage-based immigrant mothers in Taiwan. Arch. Psychiatr. Nurs..

[bib10] Chen S., Conwell Y., Vanorden K., Lu N., Fang Y., Ma Y., Chiu H. (2012). Prevalence and natural course of late-life depression in China primary care: a population based study from an urban community. J. Affect. Disord..

[bib11] Choi K.W., Jang E.H., Kim A.Y., Kim H., Park M.J., Byun S., Jeon H.J. (2021). Predictive inflammatory biomarkers for change in suicidal ideation in major depressive disorder and panic disorder: a 12-week follow-up study. J. Psychiatr. Res..

[bib12] Cohen J., Cohen P., West S.G., Aiken L.S. (2003).

[bib13] Colasanto M., Madigan S., Korczak D.J. (2020). Depression and inflammation among children and adolescents: a meta-analysis. J. Affect. Disord..

[bib14] Creaven A.M., Higgins N.M., Ginty A.T., Gallagher S. (2020). Social support, social participation, and cardiovascular reactivity to stress in the midlife in the united states (MIDUS) study. Biol. Psychol..

[bib15] Dam A.E., de Vugt M.E., Klinkenberg I.P., Verhey F.R., van Boxtel M.P. (2016). A systematic review of social support interventions for caregivers of people with dementia: are they doing what they promise?. Maturitas.

[bib16] Dienberg Love G., Seeman T.E., Weinstein M., Ryff C.D. (2010). Bioindicators in the MIDUS national study: protocol, measures, sample, and comparative context. J. Aging Health.

[bib17] Eisman A.B., Stoddard S.A., Heinze J., Caldwell C.H., Zimmerman M.A. (2015). Depressive symptoms, social support, and violence exposure among urban youth: a longitudinal study of resilience. Dev. Psychol..

[bib18] Elliot A.J., Heffner K.L., Mooney C.J., Moynihan J.A., Chapman B.P. (2018). Social relationships and inflammatory markers in the MIDUS cohort: the role of age and gender differences. J. Aging Health.

[bib19] Fatemian H., Moslemi H., Hosseini Y., Moshfeghinia R. (2024). C-reactive protein (CRP) level in depressed patients with suicidal behavior: a systematic review and meta-analysis. J. Affect. Disord..

[bib20] Felger J.C., Haroon E., Patel T.A., Goldsmith D.R., Wommack E.C., Woolwine B.J., Miller A.H. (2020). What does plasma CRP tell us about peripheral and central inflammation in depression?. Mol. Psychiatr..

[bib21] Fitzgerald M., Morgan A.A. (2022). Childhood maltreatment and provision of support and strain to family relationships in adulthood: the role of social anxious and depressive symptoms. J. Soc. Pers. Relat..

[bib22] Frank P., Kaushal A., Poole L., Lawes S., Chalder T., Cadar D. (2019). Systemic low-grade inflammation and subsequent depressive symptoms: is there a mediating role of physical activity?. Brain Behav. Immun..

[bib23] Franklyn S.I., Stewart J., Beaurepaire C., Thaw E., McQuaid R.J. (2022). Developing symptom clusters: linking inflammatory biomarkers to depressive symptom profiles. Transl. Psychiatry.

[bib24] Funder D.C., Ozer D.J. (2019). Evaluating effect size in psychological research: sense and nonsense. Adv. Methods Pract. Psychol. Sci..

[bib25] Futtrup J., Margolinsky R., Benros M.E., Moos T., Routhe L.J., Rungby J., Krogh J. (2020). Blood-brain barrier pathology in patients with severe mental disorders: a systematic review and meta-analysis of biomarkers in case-control studies. Brain Behavior Immunity Health.

[bib26] Golanov E.V., Sharpe M.A., Regnier-Golanov A.S., Del Zoppo G.J., Baskin D.S., Britz G.W. (2019). Fibrinogen chains intrinsic to the brain. Front. Neurosci..

[bib27] Guevara J.E., Murdock K.W. (2019). High social strain and physical health: examining the roles of anxious arousal, body mass index, and inflammation. Psychoneuroendocrinology.

[bib28] Hainmueller J., Mummolo J., Xu Y. (2018). How much should we trust estimates from multiplicative interaction models? Simple tools to improve empirical practice. Polit. Anal..

[bib29] Hassamal S. (2023). Chronic stress, neuroinflammation, and depression: an overview of pathophysiological mechanisms and emerging anti-inflammatories. Front. Psychiatr..

[bib30] Henneghan A., Wright M.L., Bourne G., Sales A.C. (2021). A cross-sectional exploration of cytokine-symptom networks in breast cancer survivors using network analysis. Can. J. Nurs. Res..

[bib31] Horowitz M.A., Zunszain P.A. (2015). Neuroimmune and neuroendocrine abnormalities in depression: two sides of the same coin. Ann. N. Y. Acad. Sci..

[bib32] Hughes S., Jaremka L.M., Alfano C.M., Glaser R., Povoski S.P., Lipari A.M., Kiecolt-Glaser J.K. (2014). Social support predicts inflammation, pain, and depressive symptoms: longitudinal relationships among breast cancer survivors. Psychoneuroendocrinology.

[bib33] Hunt C., Macedo E.C.T., Suchting R., de Dios C., Cuellar Leal V.A., Soares J.C., Selvaraj S. (2020). Effect of immune activation on the kynurenine pathway and depression symptoms - a systematic review and meta-analysis. Neurosci. Biobehav. Rev..

[bib34] Hunt G.E., Malhi G.S., Lai H.M.X., Cleary M. (2020). Prevalence of comorbid substance use in major depressive disorder in community and clinical settings, 1990-2019: systematic review and meta-analysis. J. Affect. Disord..

[bib35] Ignacio Z.M., da Silva R.S., Plissari M.E., Quevedo J., Reus G.Z. (2019). Physical exercise and neuroinflammation in major depressive disorder. Mol. Neurobiol..

[bib36] Imai K., Keele L., Tingley D. (2010). A general approach to causal mediation analysis. Psychol. Methods.

[bib37] Iob E., Kirschbaum C., Steptoe A. (2020). Persistent depressive symptoms, HPA-axis hyperactivity, and inflammation: the role of cognitive-affective and somatic symptoms. Mol. Psychiatr..

[bib38] Irwin J.R., McClelland G.H. (2001). Misleading heuristics and moderated multiple regression models. J. Mark. Res..

[bib39] Kane H.S., Krizan Z. (2021). Sleep, emotional supportiveness, and socially straining behavior: a multidimensional approach. Sleep Health.

[bib40] Karazsia B.T., Berlin K.S., Armstrong B., Janicke D.M., Darling K.E. (2014). Integrating mediation and moderation to advance theory development and testing. J. Pediatr. Psychol..

[bib41] Kessler R.C., Andrews G., Mroczek D., Ustun B., Wittchen H.-U. (1998). The world health organization composite international diagnostic interview - short-form (CIDI-SF). Int. J. Methods Psychiatr. Res..

[bib42] Kleih T.S., Entringer S., Scholaske L., Kathmann N., DePunder K., Heim C.M., Buss C. (2022). Exposure to childhood maltreatment and systemic inflammation across pregnancy: the moderating role of depressive symptomatology. Brain Behav. Immun..

[bib43] Koo J., Marangell L.B., Nakamura M., Armstrong A., Jeon C., Bhutani T., Wu J.J. (2017). Depression and suicidality in psoriasis: review of the literature including the cytokine theory of depression. J. Eur. Acad. Dermatol. Venereol..

[bib44] Krogh J., Benros M.E., Jorgensen M.B., Vesterager L., Elfving B., Nordentoft M. (2014). The association between depressive symptoms, cognitive function, and inflammation in major depression. Brain Behav. Immun..

[bib45] Kuhlmann S.L., Arolt V., Haverkamp W., Martus P., Strohle A., Waltenberger J., Muller-Nordhorn, J (2019). Prevalence, 12-month prognosis, and clinical management need of depression in coronary heart disease patients: a prospective cohort study. Psychother. Psychosom..

[bib46] Kushner I., Mackiewicz A., Mackiewicz A., Kushner I., Baumann H. (2020). Acute Phase Proteins: Molecular Biology, Biochemistry, and Clinical Applications.

[bib47] Lee C., Min S.H., Niitsu K. (2023). Nonlinear relationship between C-reactive protein and depression among obese middle-aged adults. Nurs. Res..

[bib48] Lee C., Whooley M.A. (2023). Networks of C-reactive protein and depression symptoms in patients with stable coronary heart disease: findings from the heart and soul study. Int. J. Methods Psychiatr. Res..

[bib49] Lei A.A., Phang V.W.X., Lee Y.Z., Kow A.S.F., Tham C.L., Ho Y.C., Lee M.T. (2025). Chronic stress-associated depressive disorders: the impact of HPA axis dysregulation and neuroinflammation on the hippocampus-A mini review. Int. J. Mol. Sci..

[bib50] Loh W.W., Moerkerke B., Loeys T., Vansteelandt S. (2022). Nonlinear mediation analysis with high-dimensional mediators whose causal structure is unknown. Biometrics.

[bib51] Luan X., Cheng H., Chen Y., Cheng L., Zhou S., Song J., He J. (2020). High levels of plasma fibrinogen and prothrombin time are related to post-stroke emotional impairment. Brain Res..

[bib52] Lutz W., Schaffrath J., Eberhardt S.T., Hehlmann M.I., Schwartz B., Deisenhofer A.K., Moggia D. (2024). Precision mental health and data-informed decision support in psychological therapy: an example. Adm. Pol. Ment. Health.

[bib53] Mac Giollabhui N., Ng T.H., Ellman L.M., Alloy L.B. (2021). The longitudinal associations of inflammatory biomarkers and depression revisited: systematic review, meta-analysis, and meta-regression. Mol. Psychiatr..

[bib54] Mahendra A., Haque A., Prabakaran P., Mackness B.C., Fuller T.P., Liu X., Chowdhury P.S. (2022). Honing-in antigen-specific cells during antibody discovery: a user-friendly process to mine a deeper repertoire. Commun. Biol..

[bib55] Marshall J.S., Warrington R., Watson W., Kim H.L. (2018). An introduction to immunology and immunopathology. Allergy Asthma Clin. Immunol..

[bib56] Mayer M. (2024). missRanger: fast imputation of missing values. https://CRAN.R-project.org/package=missRanger.

[bib57] Medina-Rodriguez E.M., Beurel E. (2022). Blood brain barrier and inflammation in depression. Neurobiol. Dis..

[bib58] Milaneschi Y., Bandinelli S., Penninx B.W., Vogelzangs N., Corsi A.M., Lauretani F., Ferrucci L. (2011). Depressive symptoms and inflammation increase in a prospective study of older adults: a protective effect of a healthy (Mediterranean-style) diet. Mol. Psychiatr..

[bib59] Moriarity D.P., van Borkulo C., Alloy L.B. (2021). Inflammatory phenotype of depression symptom structure: a network perspective. Brain Behav. Immun..

[bib60] Nakamura J.S., Kim E.S., Rentscher K.E., Bower J.E., Kuhlman K.R. (2022). Early-life stress, depressive symptoms, and inflammation: the role of social factors. Aging Ment. Health.

[bib61] Niles A.N., Smirnova M., Lin J., O'Donovan A. (2018). Gender differences in longitudinal relationships between depression and anxiety symptoms and inflammation in the health and retirement study. Psychoneuroendocrinology.

[bib62] Oenning N.S.X., Ziegelmann P.K., Goulart B.N.G., Niedhammer I. (2018). Occupational factors associated with major depressive disorder: a Brazilian population-based study. J. Affect. Disord..

[bib63] Orazani S.N., Reynolds K.J., Osborne H. (2023). What works and why in interventions to strengthen social cohesion: a systematic review. J. Appl. Soc. Psychol..

[bib64] Orsolini L., Pompili S., Tempia Valenta S., Salvi V., Volpe U. (2022). C-reactive protein as a biomarker for major depressive disorder?. Int. J. Mol. Sci..

[bib65] Osimo E.F., Pillinger T., Rodriguez I.M., Khandaker G.M., Pariante C.M., Howes O.D. (2020). Inflammatory markers in depression: a meta-analysis of mean differences and variability in 5,166 patients and 5,083 controls. Brain Behav. Immun..

[bib66] Ospina L.H., Beck-Felts K., Ifrah C., Lister A., Messer S., Russo S.J., Kimhy D. (2022). Inflammation and emotion regulation: findings from the MIDUS II study. Brain Behavior Immunity Health.

[bib67] Patel S., Govindarajan V., Chakravarty S., Dubey N. (2024). From blood to brain: exploring the role of fibrinogen in the pathophysiology of depression and other neurological disorders. Int. Immunopharmacol..

[bib68] Pearl J. (2014). Interpretation and identification of causal mediation. Psychol. Methods.

[bib69] Petersen M.A., Ryu J.K., Akassoglou K. (2018). Fibrinogen in neurological diseases: mechanisms, imaging and therapeutics. Nat. Rev. Neurosci..

[bib70] Piers T.M., East E., Villegas-Llerena C., Sevastou I.G., Matarin M., Hardy J., Pocock J.M. (2018). Soluble fibrinogen triggers non-cell autonomous ER stress-mediated microglial-induced neurotoxicity. Front. Cell. Neurosci..

[bib71] Pieters M., Wolberg A.S. (2019). Fibrinogen and fibrin: an illustrated review. Res. Practice Thrombosis Haemostasis.

[bib72] Quinn M.E., Stanton C.H., Slavich G.M., Joormann J. (2020). Executive control, cytokine reactivity to social stress, and depressive symptoms: testing the social signal transduction theory of depression. Stress.

[bib73] R Core Team (2024). https://www.R-project.org/.

[bib74] Roest A.M., de Vries Y.A., Al-Hamzawi A., Alonso J., Ayinde O.O., Bruffaerts R., collaborators, W. H. O. W. M. H. S. (2021). Previous disorders and depression outcomes in individuals with 12-month major depressive disorder in the world mental health surveys. Epidemiol. Psychiatr. Sci..

[bib75] Rook K.S. (1984). The negative side of social interaction: impact on psychological well-being. J. Pers. Soc. Psychol..

[bib76] Rosenthal R. (1994).

[bib77] Rueger S.Y., Malecki C.K., Pyun Y., Aycock C., Coyle S. (2016). A meta-analytic review of the association between perceived social support and depression in childhood and adolescence. Psychol. Bull..

[bib78] Ryff C., Almeida D., Ayanian J., Binkley N., Carr D.S., Coe C., Williams D. (2019). *Midlife in the United States (MIDUS 3), 2013-2014* inter-university consortium for political and social research.

[bib79] Ryff C., Almeida D.M., Ayanian J., Carr D.S., Cleary P.D., Coe C., Williams D. (2017). *Midlife in the United States (MIDUS 2), 2004-2006* inter-university consortium for political and social research.

[bib80] Ryff C.D., Seeman T., Weinstein M. (2019). *Midlife in the United States (MIDUS 2): Biomarker project, 2004-2009* inter-university consortium for political and social research.

[bib81] Sforzini L., Cattaneo A., Ferrari C., Turner L., Mariani N., Enache D., Pariante C.M. (2023). Higher immune-related gene expression in major depression is independent of CRP levels: results from the BIODEP study. Transl. Psychiatry.

[bib82] Shieh G. (2010). Sample size determination for confidence intervals of interaction effects in moderated multiple regression with continuous predictor and moderator variables. Behav. Res. Methods.

[bib83] Slavich G.M., Irwin M.R. (2014). From stress to inflammation and major depressive disorder: a social signal transduction theory of depression. Psychol. Bull..

[bib84] Stekhoven D.J., Buhlmann P. (2012). MissForest–non-parametric missing value imputation for mixed-type data. Bioinformatics.

[bib85] Straka K., Tran M.L., Millwood S., Swanson J., Kuhlman K.R. (2020). Aging as a context for the role of inflammation in depressive symptoms. Front. Psychiatr..

[bib86] Tang F., Ishwaran H. (2017). Random forest missing data algorithms. Stat. Anal. Data Min..

[bib87] Taylor S.E., Friedman H.S. (2011).

[bib88] Teo A.R., Choi H., Valenstein M. (2013). Social relationships and depression: ten-year follow-up from a nationally representative study. PLoS One.

[bib89] Toussaint L.L., Moriarity D.P., Kamble S., Williams D.R., Slavich G.M. (2022). Inflammation and depression symptoms are most strongly associated for black adults. Brain Behavior Immunity Health.

[bib90] Uchino B.N., Trettevik R., Kent de Grey R.G., Cronan S., Hogan J., Baucom B.R.W. (2018). Social support, social integration, and inflammatory cytokines: a meta-analysis. Health Psychol..

[bib91] Vandeleur C.L., Fassassi S., Castelao E., Glaus J., Strippoli M.F., Lasserre A.M., Preisig M. (2017). Prevalence and correlates of DSM-5 major depressive and related disorders in the community. Psychiatry Res..

[bib92] VanderWeele T.J., Tchetgen Tchetgen E.J. (2017). Mediation analysis with time varying exposures and mediators. J. R Stat. Soc. Ser. B Stat. Methodol..

[bib93] Weinstein M., Ryff C.D., Seeman T.E. (2019). *Midlife in the United States (MIDUS refresher): Biomarker project, 2012-2016* inter-university consortium for political and social research.

[bib94] Wieling M. (2018). Analyzing dynamic phonetic data using generalized additive mixed modeling: a tutorial focusing on articulatory differences between L1 and L2 speakers of English. J. Phonetics.

[bib95] Witzel D.D., Bhat A.C., Graham-Engeland J.E., Almeida D.M. (2025). Age and inflammation: insights on "age three ways" from midlife in the United States study. Brain Behav. Immun..

[bib96] Wium-Andersen M.K., Orsted D.D., Nordestgaard B.G. (2013). Association between elevated plasma fibrinogen and psychological distress, and depression in 73,367 individuals from the general population. Mol. Psychiatr..

[bib97] Wood S.N. (2004). Stable and efficient multiple smoothing parameter estimation for generalized additive models. J. Am. Stat. Assoc..

[bib98] Wood S.N. (2011). Fast stable restricted maximum likelihood and marginal likelihood estimation of semiparametric generalized linear models. J. Roy. Stat. Soc. B.

[bib99] Wood S.N. (2017).

[bib100] Wood S.N., Goude Y., Shaw S. (2015). Generalized additive models for large data sets. J. Roy. Stat. Soc. C Appl. Stat..

[bib101] Yang Y.C., Schorpp K., Harris K.M. (2014). Social support, social strain and inflammation: evidence from a national longitudinal study of U.S. adults. Soc. Sci. Med..

[bib102] Yuan K.H., Cheng Y., Maxwell S. (2014). Moderation analysis using a two-level regression model. Psychometrika.

[bib103] Zainal N.H., Newman M.G. (2021). Increased inflammation predicts nine-year change in major depressive disorder diagnostic status. J. Abnorm. Psychol..

[bib104] Zainal N.H., Newman M.G. (2022). Depression and worry symptoms predict future executive functioning impairment via inflammation. Psychol. Med..

[bib105] Zainal N.H., Newman M.G. (2022). Inflammation mediates depression and generalized anxiety symptoms predicting executive function impairment after 18 years. J. Affect. Disord..

[bib106] Zainal N.H., Newman M.G. (2023). Prospective network analysis of proinflammatory proteins, lipid markers, and depression components in midlife community women. Psychol. Med..

[bib107] Zavos H.M.S., Zunszain P.A., Jayaweera K., Powell T.R., Chatzivasileiadou M., Harber-Aschan L., Rijsdijk F. (2022). Relationship between CRP and depression: a genetically sensitive study in Sri Lanka. J. Affect. Disord..

[bib108] Zhai S., Qu Y., Zhang D., Li T., Xie Y., Wu X., Tao S. (2022). Depressive symptoms predict longitudinal changes of chronic inflammation at the transition to adulthood. Front. Immunol..

