# Supplementary material for: The inflammation-depression link: How social networks buffer or exacerbate risk
Source: Brain Behav Immun Health. 2025 Jul 4;48:101052. doi: 10.1016/j.bbih.2025.101052 (PMC12274883; doi:10.1016/j.bbih.2025.101052)
Supplement: Multimedia component 1 [file mmc1.docx]

# The Inflammation-Depression Link: How Social Networks Buffer or Exacerbate Risk

# Online Supplemental Materials (OSM)

## Availability of Data and Materials

The present study is differentiated from prior studies that similarly used the Midlife Development in the United States (MIDUS) dataset (Ryff et al., 2019a; Ryff et al., 2017; Ryff et al., 2019b). It holistically studies the moderating effects of both social support and strain dimensions on the associations between baseline C-reactive protein (CRP) and fibrinogen levels (proinflammatory activity markers) and nine-year major depressive disorder (MDD) severity. Although earlier research has studied the correlations among proinflammatory activity, social relationship quality, and MDD severity, they typically evaluated these variables independently or did not account for their potential interactive effects across long durations. For instance, Yang et al. (2014) examined the degree to which social support and strain dimensions were associated with inflammation levels but did not test their combined effects on future MDD severity. Likewise, Kaveladze et al. (2022) investigated the longitudinal relations among depressive symptoms, social relationship quality, and inflammation levels. However, this study did not test how social support and strain dimensions might moderate the trajectory from baseline inflammation levels to future MDD severity. The present study harnessed both multiple linear regression and generalized additive modeling to test linear and non-linear interactions. Therefore, the current study offers new knowledge on how interpersonal factors could magnify or minimize the effect of persistently high inflammation levels on long-term MDD severity. This unified framework aligns with the social signal transduction model of depression, providing a subtle comprehension of the biopsychosocial and distal risk factors of increased MDD severity.

Table S1

*Descriptive statistics of study variables*

|  |  | Total (*N* = 1,054) | |  |
| --- | --- | --- | --- | --- |
| Continuous variables |  | *M* | (*SD*) |  |
| Age (years) |  | 55.19 | (11.81) |  |
| W1 Social support |  | 46.86 | (10.08) |  |
| W1 Social strain |  | 30.51 | (12.73) |  |
| W1 MDD severity |  | 0.62 | (1.87) |  |
| W2 MDD severity |  | 0.51 | (1.72) |  |
| W1 Fibrinogen |  | 5.21 | (0.43) |  |
| W1 CRP |  | 0.17 | (0.38) |  |
| Categorical variables |  | *n* | (%) |  |
| Gender |  |  |  |  |
| Male |  | 477 | (45.30) |  |
| Female |  | 577 | (54.70) |  |
| Racial identity |  |  |  |  |
| Declined to disclose |  | 30 | (2.85) |  |
| Multiracial |  | 8 | (0.76) |  |
| White |  | 961 | (91.20) |  |
| African American |  | 29 | (2.75) |  |
| Native American |  | 5 | (0.47) |  |
| Asian |  | 2 | (0.19) |  |
| Other |  | 19 | (1.80) |  |
| Education level |  |  |  |  |
| College education and above |  | 465 | (44.10) |  |
| High school |  | 238 | (22.60) |  |
| No high school degree |  | 51 | (4.84) |  |
| Some college |  | 300 | (28.50) |  |
| W1 MDD status |  |  |  |  |
| Present |  | 123 | (11.70) |  |
| Absent |  | 931 | (88.30) |  |
| W2 MDD status |  |  |  |  |
| Present |  | 96 | (9.11) |  |
| Absent |  | 958 | (90.89) |  |

*Note.* *d*, Cohen’s *d* effect sizes; W1, wave 1 (2004–2006); W2, wave 2 (2013–2014); MDD, major depressive disorder; CRP, C-reactive protein. Statistically significant between-group differences are depicted by bold *p*-values.

Table S2

*Between-group differences in study variables among dropouts and completers*

|  |  | Dropout (*n* = 178) | |  | Completer (*n* = 876) | |  | *p* |  | *d* |
| --- | --- | --- | --- | --- | --- | --- | --- | --- | --- | --- |
| Continuous variables |  | *M* | (*SD*) |  | *M* | (*SD*) |  |  |  |  |
| Age (years) |  | 59.27 | (14.96) |  | 54.36 | (10.89) |  | **.000** |  | 0.38 |
| W1 Social support |  | 47.79 | (11.48) |  | 46.73 | (9.82) |  | .693 |  | 0.10 |
| W1 Social strain |  | 32.85 | (13.39) |  | 29.94 | (12.54) |  | **.015** |  | 0.22 |
| W1 MDD severity |  | 0.67 | (1.91) |  | 0.60 | (1.86) |  | .507 |  | 0.04 |
| W2 MDD severity |  | 0.34 | (1.42) |  | 0.48 | (1.70) |  | .291 |  | 0.09 |
| W1 Fibrinogen |  | 5.28 | (0.45) |  | 5.19 | (0.42) |  | **.012** |  | 0.21 |
| W1 CRP |  | 0.65 | (0.85) |  | 0.56 | (0.80) |  | .178 |  | 0.10 |
| Categorical variables |  | *n* | (%) |  | *n* | (%) |  | *p* |  | χ^2^ |
| Gender |  |  |  |  |  |  |  | .189 |  | 0.08 |
| Male |  | 89 | (50.00) |  | 388 | (44.30) |  |  |  |  |
| Female |  | 89 | (50.00) |  | 488 | (55.70) |  |  |  |  |
| Racial identity |  |  |  |  |  |  |  | **.006** |  | 0.17 |
| Declined to disclose |  | 16 | (1.83) |  | 14 | (7.87) |  |  |  |  |
| Multiracial |  | 7 | (0.80) |  | 1 | (0.56) |  |  |  |  |
| White |  | 808 | (92.20) |  | 153 | (86.00) |  |  |  |  |
| African American |  | 24 | (2.74) |  | 5 | (2.81) |  |  |  |  |
| Native American |  | 3 | (0.34) |  | 0 | (0.00) |  |  |  |  |
| Asian |  | 2 | (0.23) |  | 2 | (1.12) |  |  |  |  |
| Other |  | 16 | (1.83) |  | 3 | (1.69) |  |  |  |  |
| Education level |  |  |  |  |  |  |  | .353 |  | 0.06 |
| College education and above |  | 73 | (41.00) |  | 392 | (44.70) |  |  |  |  |
| High school |  | 42 | (23.60) |  | 196 | (22.40) |  |  |  |  |
| No high school degree |  | 13 | (7.30) |  | 38 | (4.34) |  |  |  |  |
| Some college |  | 50 | (28.10) |  | 250 | (28.50) |  |  |  |  |
| W1 MDD status |  |  |  |  |  |  |  | .340 |  | 0.91 |
| Present |  | 25 | (14.00) |  | 98 | (11.20) |  |  |  |  |
| Absent |  | 153 | (86.00) |  | 778 | (88.80) |  |  |  |  |
| W2 MDD status |  |  |  |  |  |  |  | .438 |  | 0.60 |
| Present |  | 13 | (7.30) |  | 83 | (9.47) |  |  |  |  |
| Absent |  | 165 | (92.70) |  | 793 | (90.53) |  |  |  |  |

*Note.* *d*, Cohen’s *d* effect sizes; W1, wave 1 (2004–2006); W2, wave 2 (2013–2014); MDD, major depressive disorder; CRP, C-reactive protein. Statistically significant between-group differences are depicted by bold *p*-values.

Table S3

*Multiple regression analysis of W1 fibrinogen moderating the effects of W1 social support dimensions on W2 MDD severity adjusting for age*

| Parameter estimate | *b* | β | (*SE*) | *t* | *p* | *d* |
| --- | --- | --- | --- | --- | --- | --- |
| Intercept | -5.391 | – | (3.080) | -1.751 | .080 | -0.108 |
| W1 MDD severity | 0.270 | 0.294 | (0.027) | 9.969 | .000 | 0.616 |
| W1 Social support | 0.258 | 1.513 | (0.091) | 2.819 | .005 | 0.174 |
| W1 Fibrinogen | 1.289 | 0.322 | (0.588) | 2.190 | .029 | 0.135 |
| W1 Social strain | -0.260 | -1.926 | (0.071) | -3.682 | .000 | -0.228 |
| W1 Age | -0.010 | -0.068 | (0.004) | -2.295 | .022 | -0.142 |
| W1 Social support x W1 Fibrinogen | -0.055 | -1.836 | (0.018) | -3.118 | .002 | -0.193 |
| W1 Social strain x W1 Fibrinogen | 0.054 | 2.193 | (0.014) | 4.025 | .000 | 0.249 |

*Note.* W1, wave 1; W2, wave 2; MDD, major depressive disorder. Any ‘–’ indicated that the specific parameter values could not be estimated.

Table S4

*Multiple regression analysis of W1 fibrinogen moderating the effects of W1 social support dimensions on W2 MDD severity adjusting for gender*

| Parameter estimate | *b* | β | (*SE*) | *t* | *p* | *d* |
| --- | --- | --- | --- | --- | --- | --- |
| Intercept | -5.617 | – | (3.081) | -1.823 | .069 | -0.113 |
| W1 MDD severity | 0.268 | 0.293 | (0.027) | 9.871 | .000 | 0.610 |
| W1 Social support | 0.256 | 1.503 | (0.092) | 2.797 | .005 | 0.173 |
| W1 Fibrinogen | 1.218 | 0.305 | (0.589) | 2.068 | .039 | 0.128 |
| W1 Social strain | -0.266 | -1.968 | (0.070) | -3.766 | .000 | -0.233 |
| W1 Gender | 0.193 | 0.056 | (0.101) | 1.906 | .057 | 0.118 |
| W1 Social support x W1 Fibrinogen | -0.055 | -1.856 | (0.018) | -3.151 | .002 | -0.195 |
| W1 Social strain x W1 Fibrinogen | 0.056 | 2.253 | (0.013) | 4.143 | .000 | 0.256 |

*Note.* W1, wave 1; W2, wave 2; MDD, major depressive disorder. Any ‘–’ indicated that the specific parameter values could not be estimated.

Table S5

*Multiple regression analysis of W1 fibrinogen moderating the effects of W1 social support dimensions on W2 MDD severity adjusting for race*

| Parameter estimate | *b* | β | (*SE*) | *t* | *p* | *d* |
| --- | --- | --- | --- | --- | --- | --- |
| Intercept | -5.627 | – | (3.089) | -1.821 | .069 | -0.113 |
| W1 MDD severity | 0.275 | 0.300 | (0.027) | 10.169 | .000 | 0.629 |
| W1 Social support | 0.265 | 1.553 | (0.092) | 2.884 | .004 | 0.178 |
| W1 Fibrinogen | 1.266 | 0.317 | (0.591) | 2.141 | .033 | 0.132 |
| W1 Social strain | -0.273 | -2.024 | (0.071) | -3.869 | .000 | -0.239 |
| W1 White vs. Non-White | -0.011 | -0.005 | (0.062) | -0.172 | .863 | -0.011 |
| W1 Social support x W1 Fibrinogen | -0.057 | -1.902 | (0.018) | -3.220 | .001 | -0.199 |
| W1 Social strain x W1 Fibrinogen | 0.057 | 2.312 | (0.014) | 4.248 | .000 | 0.263 |

*Note.* W1, wave 1; W2, wave 2; MDD, major depressive disorder. Any ‘–’ indicated that the specific parameter values could not be estimated.

Table S6

*Multiple regression analysis of W1 fibrinogen moderating the effects of W1 social support dimensions on W2 MDD severity adjusting for education level*

| Parameter estimate | *b* | β | (*SE*) | *t* | *p* | *d* |
| --- | --- | --- | --- | --- | --- | --- |
| Intercept | -5.508 | – | (3.082) | -1.787 | .074 | -0.111 |
| W1 MDD severity | 0.273 | 0.298 | (0.027) | 10.097 | .000 | 0.624 |
| W1 Social support | 0.265 | 1.553 | (0.092) | 2.891 | .004 | 0.179 |
| W1 Fibrinogen | 1.232 | 0.308 | (0.589) | 2.093 | .037 | 0.129 |
| W1 Social strain | -0.277 | -2.053 | (0.070) | -3.932 | .000 | -0.243 |
| W1 College education vs. High school | -0.078 | -0.019 | (0.127) | -0.611 | .541 | -0.038 |
| W1 College education vs. No high school degree | 0.477 | 0.060 | (0.235) | 2.026 | .043 | 0.125 |
| W1 College education vs. Some college | 0.103 | 0.027 | (0.118) | 0.871 | .384 | 0.054 |
| W1 Social support x W1 Fibrinogen | -0.056 | -1.896 | (0.018) | -3.220 | .001 | -0.199 |
| W1 Social strain x W1 Fibrinogen | 0.058 | 2.335 | (0.013) | 4.298 | .000 | 0.266 |

*Note.* W1, wave 1; W2, wave 2; MDD, major depressive disorder. Any ‘–’ indicated that the specific parameter values could not be estimated.

Table S7

*Multiple regression analysis of W1 fibrinogen moderating the effects of W1 social support dimensions on W2 MDD severity adjusting for childhood trauma*

| Parameter estimate | *b* | β | (*SE*) | *t* | *p* | *d* |
| --- | --- | --- | --- | --- | --- | --- |
| Intercept | -7.247 | – | (3.113) | -2.328 | .020 | -0.144 |
| W1 MDD severity | 0.265 | 0.289 | (0.027) | 9.774 | .000 | 0.604 |
| W1 Social support | 0.256 | 1.500 | (0.091) | 2.803 | .005 | 0.173 |
| W1 Fibrinogen | 1.289 | 0.322 | (0.587) | 2.195 | .028 | 0.136 |
| W1 Social strain | -0.256 | -1.895 | (0.070) | -3.633 | .000 | -0.225 |
| W1 Childhood trauma | 0.024 | 0.092 | (0.007) | 3.208 | .001 | 0.198 |
| W1 Social support x W1 Fibrinogen | -0.055 | -1.849 | (0.017) | -3.149 | .002 | -0.195 |
| W1 Social strain x W1 Fibrinogen | 0.054 | 2.181 | (0.013) | 4.020 | .000 | 0.249 |

*Note.* W1, wave 1; W2, wave 2; MDD, major depressive disorder. Any ‘–’ indicated that the specific parameter values could not be estimated.

Table S8

*Multiple regression analysis of W1 fibrinogen moderating the effects of W1 social support dimensions on W2 MDD severity adjusting for GAD symptoms*

| Parameter estimate | *b* | β | (*SE*) | *t* | *p* | *d* |
| --- | --- | --- | --- | --- | --- | --- |
| Intercept | -6.394 | NA | (3.039) | -2.104 | .036 | -0.130 |
| W1 MDD severity | 0.232 | 0.253 | (0.028) | 8.418 | .000 | 0.521 |
| W1 Social support | 0.265 | 1.557 | (0.090) | 2.943 | .003 | 0.182 |
| W1 Fibrinogen | 1.292 | 0.323 | (0.580) | 2.227 | .026 | 0.138 |
| W1 Social strain | -0.268 | -1.985 | (0.069) | -3.860 | .000 | -0.239 |
| W1 GAD symptoms | 0.035 | 0.176 | (0.006) | 5.881 | .000 | 0.364 |
| W1 Social support x W1 Fibrinogen | -0.055 | -1.864 | (0.017) | -3.214 | .001 | -0.199 |
| W1 Social strain x W1 Fibrinogen | 0.055 | 2.227 | (0.013) | 4.161 | .000 | 0.257 |

*Note.* W1, wave 1; W2, wave 2; MDD, major depressive disorder; GAD, generalized anxiety disorder. Any ‘–’ indicated that the specific parameter values could not be estimated.

Table S9

*Multiple regression analysis of W1 fibrinogen moderating the effects of W1 social support dimensions on W2 MDD severity adjusting for panic symptoms*

| Parameter estimate | *b* | β | (*SE*) | *t* | *p* | *d* |
| --- | --- | --- | --- | --- | --- | --- |
| Intercept | -5.260 | NA | (3.070) | -1.713 | .087 | -0.106 |
| W1 MDD severity | 0.243 | 0.265 | (0.028) | 8.568 | .000 | 0.530 |
| W1 Social support | 0.245 | 1.438 | (0.091) | 2.686 | .007 | 0.166 |
| W1 Fibrinogen | 1.180 | 0.295 | (0.587) | 2.012 | .045 | 0.124 |
| W1 Social strain | -0.253 | -1.874 | (0.070) | -3.596 | .000 | -0.222 |
| W1 Panic symptoms | 0.155 | 0.107 | (0.045) | 3.479 | .001 | 0.215 |
| W1 Social support x W1 Fibrinogen | -0.052 | -1.763 | (0.017) | -3.001 | .003 | -0.186 |
| W1 Social strain x W1 Fibrinogen | 0.053 | 2.137 | (0.013) | 3.937 | .000 | 0.243 |

*Note.* W1, wave 1; W2, wave 2; MDD, major depressive disorder. Any ‘–’ indicated that the specific parameter values could not be estimated.

Table S10

*Multiple regression analysis of W1 CRP moderating the effects of W1 social support dimensions on W2 MDD severity adjusting for age*

| Parameter estimate | *b* | β | (*SE*) | *t* | *p* | *d* |
| --- | --- | --- | --- | --- | --- | --- |
| Intercept | 1.212 | – | (0.312) | 3.883 | .000 | 0.240 |
| W1 MDD severity | 0.268 | 0.292 | (0.027) | 9.830 | .000 | 0.608 |
| W1 Social support | -0.023 | -0.136 | (0.008) | -2.803 | .005 | -0.173 |
| W1 CRP | 0.038 | 0.008 | (0.654) | 0.059 | .953 | 0.004 |
| W1 Social strain | 0.019 | 0.137 | (0.007) | 2.822 | .005 | 0.174 |
| W1 Age | -0.009 | -0.060 | (0.004) | -2.016 | .044 | -0.125 |
| W1 Social support x W1 CRP | -0.017 | -0.177 | (0.019) | -0.889 | .374 | -0.055 |
| W1 Social strain x W1 CRP | 0.033 | 0.247 | (0.015) | 2.253 | .024 | 0.139 |

*Note.* W1, wave 1; CRP, C-reactive protein; W2, wave 2; MDD, major depressive disorder. Any ‘–’ indicated that the specific parameter values could not be estimated.

Table S11

*Multiple regression analysis of W1 CRP moderating the effects of W1 social support dimensions on W2 MDD severity adjusting for gender*

| Parameter estimate | *b* | β | (*SE*) | *t* | *p* | *d* |
| --- | --- | --- | --- | --- | --- | --- |
| Intercept | 0.663 | – | (0.292) | 2.269 | .023 | 0.140 |
| W1 MDD severity | 0.264 | 0.288 | (0.027) | 9.654 | .000 | 0.597 |
| W1 Social support | -0.028 | -0.167 | (0.008) | -3.487 | .001 | -0.216 |
| W1 CRP | -0.196 | -0.043 | (0.654) | -0.299 | .765 | -0.019 |
| W1 Social strain | 0.020 | 0.149 | (0.007) | 3.086 | .002 | 0.191 |
| W1 Gender | 0.231 | 0.067 | (0.102) | 2.257 | .024 | 0.140 |
| W1 Social support x W1 CRP | -0.013 | -0.133 | (0.019) | -0.668 | .505 | -0.041 |
| W1 Social strain x W1 CRP | 0.033 | 0.250 | (0.015) | 2.286 | .022 | 0.141 |

*Note.* W1, wave 1; CRP, C-reactive protein; W2, wave 2; MDD, major depressive disorder. Any ‘–’ indicated that the specific parameter values could not be estimated.

Table S12

*Multiple regression analysis of W1 CRP moderating the effects of W1 social support dimensions on W2 MDD severity adjusting for race*

| Parameter estimate | *b* | β | (*SE*) | *t* | *p* | *d* |
| --- | --- | --- | --- | --- | --- | --- |
| Intercept | 0.882 | – | (0.282) | 3.128 | 0.002 | 0.193 |
| W1 MDD severity | 0.273 | 0.298 | (0.027) | 10.026 | 0.000 | 0.620 |
| W1 Social support | -0.026 | -0.153 | (0.008) | -3.217 | 0.001 | -0.199 |
| W1 CRP | -0.071 | -0.015 | (0.654) | -0.108 | 0.914 | -0.007 |
| W1 Social strain | 0.020 | 0.148 | (0.007) | 3.062 | 0.002 | 0.189 |
| W1 White vs. Non-White | 0.019 | 0.009 | (0.062) | 0.312 | 0.755 | 0.019 |
| W1 Social support x W1 CRP | -0.015 | -0.160 | (0.019) | -0.801 | 0.423 | -0.050 |
| W1 Social strain x W1 CRP | 0.034 | 0.257 | (0.015) | 2.342 | 0.019 | 0.145 |

*Note.* W1, wave 1; CRP, C-reactive protein; W2, wave 2; MDD, major depressive disorder. Any ‘–’ indicated that the specific parameter values could not be estimated.

Table S13

*Multiple regression analysis of W1 CRP moderating the effects of W1 social support dimensions on W2 MDD severity adjusting for education level*

| Parameter estimate | *b* | β | (*SE*) | *t* | *p* | *d* |
| --- | --- | --- | --- | --- | --- | --- |
| Intercept | 0.872 | – | 0.280 | 3.111 | .002 | 0.192 |
| W1 MDD severity | 0.271 | 0.295 | 0.027 | 9.921 | .000 | 0.614 |
| W1 Social support | -0.026 | -0.150 | 0.008 | -3.138 | .002 | -0.194 |
| W1 CRP | -0.117 | -0.026 | 0.654 | -0.178 | .858 | -0.011 |
| W1 Social strain | 0.019 | 0.141 | 0.007 | 2.904 | .004 | 0.180 |
| W1 College education vs. High school | -0.078 | -0.019 | 0.128 | -0.609 | .543 | -0.038 |
| W1 College education vs. No high school degree | 0.480 | 0.060 | 0.237 | 2.025 | .043 | 0.125 |
| W1 College education vs. Some college | 0.101 | 0.027 | 0.119 | 0.849 | .396 | 0.053 |
| W1 Social support x W1 CRP | -0.015 | -0.154 | 0.019 | -0.771 | .441 | -0.048 |
| W1 Social strain x W1 CRP | 0.035 | 0.260 | 0.015 | 2.372 | .018 | 0.147 |

*Note.* W1, wave 1; CRP, C-reactive protein; W2, wave 2; MDD, major depressive disorder. Any ‘–’ indicated that the specific parameter values could not be estimated.

Table S14

*Multiple regression analysis of W1 CRP moderating the effects of W1 social support dimensions on W2 MDD severity adjusting for childhood trauma*

| Parameter estimate | *b* | β | (*SE*) | *t* | *p* | *d* |
| --- | --- | --- | --- | --- | --- | --- |
| Intercept | -0.625 | NA | (0.537) | -1.163 | .245 | -0.072 |
| W1 MDD severity | 0.262 | 0.285 | (0.027) | 9.594 | .000 | 0.593 |
| W1 Social support | -0.027 | -0.161 | (0.008) | -3.390 | .001 | -0.210 |
| W1 CRP | -0.100 | -0.022 | (0.650) | -0.153 | .878 | -0.009 |
| W1 Social strain | 0.021 | 0.156 | (0.007) | 3.236 | .001 | 0.200 |
| W1 Childhood trauma | 0.024 | 0.095 | (0.007) | 3.300 | .001 | 0.204 |
| W1 Social support x W1 CRP | -0.013 | -0.143 | (0.019) | -0.720 | .471 | -0.045 |
| W1 Social strain x W1 CRP | 0.032 | 0.242 | (0.015) | 2.216 | .027 | 0.137 |

*Note.* W1, wave 1; CRP, C-reactive protein; W2, wave 2; MDD, major depressive disorder. Any ‘–’ indicated that the specific parameter values could not be estimated.

Table S15

*Multiple regression analysis of W1 CRP moderating the effects of W1 social support dimensions on W2 MDD severity adjusting for GAD symptoms*

| Parameter estimate | *b* | β | (*SE*) | *t* | *p* | *d* |
| --- | --- | --- | --- | --- | --- | --- |
| Intercept | 0.276 | NA | (0.290) | 0.953 | .341 | 0.059 |
| W1 MDD severity | 0.231 | 0.251 | (0.028) | 8.295 | .000 | 0.513 |
| W1 Social support | -0.020 | -0.116 | (0.008) | -2.435 | .015 | -0.151 |
| W1 CRP | 0.069 | 0.015 | (0.644) | 0.108 | .914 | 0.007 |
| W1 Social strain | 0.015 | 0.111 | (0.006) | 2.296 | .022 | 0.142 |
| W1 GAD symptoms | 0.035 | 0.173 | (0.006) | 5.727 | .000 | 0.354 |
| W1 Social support x W1 CRP | -0.017 | -0.179 | (0.019) | -0.910 | .363 | -0.056 |
| W1 Social strain x W1 CRP | 0.032 | 0.242 | (0.014) | 2.239 | .025 | 0.138 |

*Note.* W1, wave 1; CRP, C-reactive protein; W2, wave 2; MDD, major depressive disorder; GAD, generalized anxiety disorder. Any ‘–’ indicated that the specific parameter values could not be estimated.

Table S16

*Multiple regression analysis of W1 CRP moderating the effects of W1 social support dimensions on W2 MDD severity adjusting for panic symptoms*

| Parameter estimate | *b* | β | (*SE*) | *t* | *p* | *d* |
| --- | --- | --- | --- | --- | --- | --- |
| Intercept | 0.844 | – | (0.272) | 3.107 | .002 | 0.192 |
| W1 MDD severity | 0.239 | 0.261 | (0.029) | 8.364 | .000 | 0.517 |
| W1 Social support | -0.025 | -0.144 | (0.008) | -3.030 | .003 | -0.187 |
| W1 CRP | -0.082 | -0.018 | (0.649) | -0.126 | .900 | -0.008 |
| W1 Social strain | 0.018 | 0.134 | (0.007) | 2.764 | .006 | 0.171 |
| W1 Panic symptoms | 0.165 | 0.113 | (0.045) | 3.668 | .000 | 0.227 |
| W1 Social support x W1 CRP | -0.014 | -0.147 | (0.019) | -0.744 | .457 | -0.046 |
| W1 Social strain x W1 CRP | 0.032 | 0.236 | (0.015) | 2.166 | .031 | 0.134 |

*Note.* W1, wave 1; CRP, C-reactive protein; W2, wave 2; MDD, major depressive disorder. Any ‘–’ indicated that the specific parameter values could not be estimated.

References

Kaveladze, B., Diamond Altman, A., Niederhausen, M., Loftis, J. M., & Teo, A. R. (2022). Social relationship quality, depression and inflammation: A cross-cultural longitudinal study in the United States and Tokyo, Japan. *International Journal of Social Psychiatry*, *68*(2), 253-263. <https://doi.org/10.1177/0020764020981604>

Ryff, C., Almeida, D., Ayanian, J., Binkley, N., Carr, D. S., Coe, C., . . . Williams, D. (2019a). *Midlife in the United States (MIDUS 3), 2013-2014* Inter-university Consortium for Political and Social Research [distributor]. <https://doi.org/10.3886/ICPSR36346.v7>

Ryff, C., Almeida, D. M., Ayanian, J., Carr, D. S., Cleary, P. D., Coe, C., . . . Williams, D. (2017). *Midlife in the United States (MIDUS 2), 2004-2006* Inter-university Consortium for Political and Social Research [distributor]. <https://doi.org/10.3886/ICPSR04652.v7>

Ryff, C. D., Seeman, T., & Weinstein, M. (2019b). *Midlife in the United States (MIDUS 2): Biomarker Project, 2004-2009* Inter-university Consortium for Political and Social Research [distributor]. <https://doi.org/10.3886/ICPSR29282.v9>

Yang, Y. C., Schorpp, K., & Harris, K. M. (2014). Social support, social strain and inflammation: evidence from a national longitudinal study of U.S. adults. *Social Science & Medicine*, *107*, 124-135. <https://doi.org/10.1016/j.socscimed.2014.02.013>
